# Optical Coherence Tomography of Bilateral Nanophthalmos with Macular Folds and High Hyperopia

**DOI:** 10.1155/2014/173853

**Published:** 2014-08-12

**Authors:** Firat Helvacioglu, Ziya Kapran, Sadik Sencan, Murat Uyar, Ozlem Cam

**Affiliations:** Department of Ophthalmology, Maltepe University School of Medicine, Altunizade, Fahrettin Kerim Gokay Sokak No. 48 Selcuklu Konaklari, C-3/6 Uskudar, 34660 Istanbul, Turkey

## Abstract

*Importance.* There is a conflict about the content of the macular folds in nanophthalmic eyes in the literature. Our study clearly demonstrated that papillomacular folds seen in nanophthalmos or posterior microphthalmos were only composed of neurosensory retina without involvement of retinal pigment epithelium and choroid. *Observations.* This is a report of two consecutive nanophthalmic patients with macular folds at Maltepe University School of Medicine, Department of Ophthalmology, from January to June 2012. Anterior segment dimensions were near normal. The axial lengths of the eyes were short with markedly shortened posterior segment. A macular fold extending from the center of the fovea towards the optic nerve head was present in all eyes. Optic coherence tomography clearly demonstrated that folds were only composed of neurosensory retina. Binocular visual acuities and refractive errors of the cases were 0.3, 0.2 and +16.00, +15.75 diopters, respectively. *Conclusions and Relevance.* Our study proposes a surgical option to treat these folds like serous retinal detachments by showing the true content of the folds, although there is not any surgical operation accepted for this condition yet. Further studies dealing with the surgical interventions of these folds should be performed to support this option.

## 1. Introduction

Nanophthalmos is a rare condition and results from the developmental arrest of the globe in all dimensions without other major malformations [1]. Nanophthalmos is sometimes considered within the spectrum of microphthalmos. These conditions can be separated clinically since microphthalmos is usually associated with structural changes of the globe and systemic anomalies [1].

Different funduscopic appearances have been shown in nanophthalmic eyes, including pigmentary changes from retinal flecks to bone spicules, bull's eye maculopathy, macular hypoplasia, retinal and macular cysts, and papillomacular folds [2, 3].

We describe two cases of bilateral nanophthalmos with yellow pigmented macular folds and high hyperopia.

## 2. Case  1

**Figure 1:**
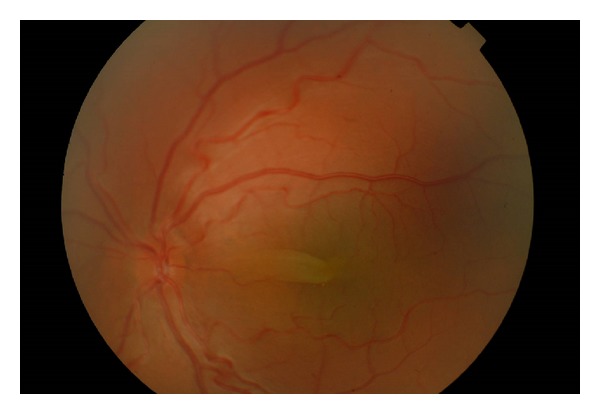
Fundus photograph of the macular folds.

**Figure 2:**
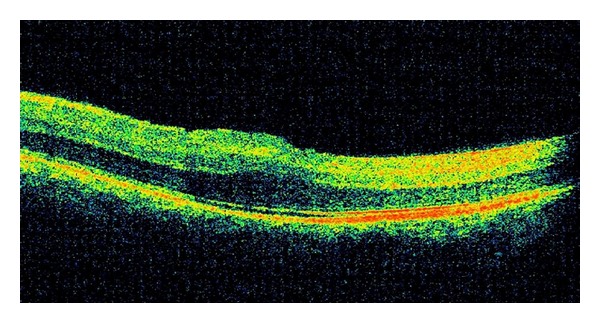
OCT image of the macular folds. The folds consist of only sensorial retina without involvement of underlying retinal pigment epithelium and choroid.

A 6-year-old male presented with best corrected visual acuity of 0.3 OU with +16.00 diopters (D) refractive errors in both eyes. Axial lengths were 14.20 mm OD and 14.50 mm OS. Horizontal and vertical corneal diameters were 9.5 mm and 9.8 mm in right eye and 9.2 mm and 9.6 mm in left eye, respectively. The intraocular pressure was measured as 16 mmHg OD, 18 mmHg OS. Pachymetry and anterior chamber angle measurements were performed by optical coherence tomography (OCT). Central corneal thicknesses of right and left eye were 527 microns and 534 microns, respectively. Temporal anterior chamber degrees of right and left eye were 32.59 degrees and 35.44 degrees, respectively. Funduscopic examination showed crowded discs with blurred margins along with horizontal macular folds emanating from the fovea and extending nasally to the optic disc (Figure 1). Horizontal and vertical papillary diameters of OD and OS were 1749 *μ*, 2282 *μ* and 1658 *μ*, 2312 *μ*, respectively. Choroidal thicknesses of OD and SO were 405 *μ* and 389 *μ*, respectively. OCT demonstrated papillomacular fold of sensorial retina without involvement of underlying retinal pigment epithelium and choroid (Figure 2).

## 3. Case  2

**Figure 3:**
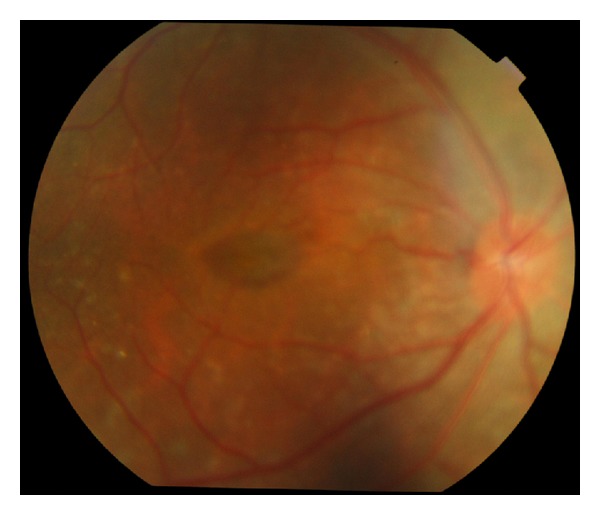
Fundus photograph of the macular folds.

**Figure 4:**
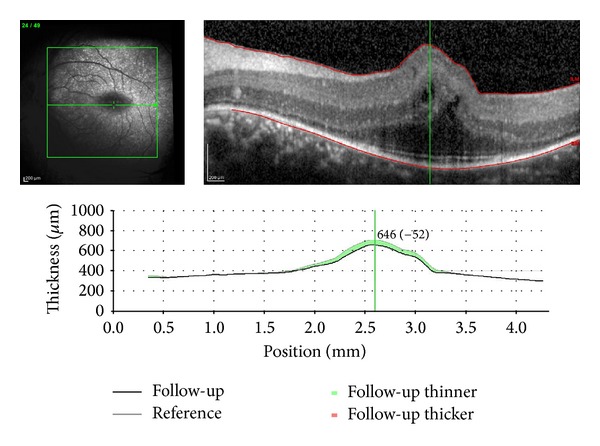
OCT image of the macular folds. The folds consist of only sensorial retina without involvement of underlying retinal pigment epithelium and choroid.

A 30-year-old male presented with best corrected visual acuity of 0.2 OU with +1.50 D at 125 cylindrical degree refractive errors in OD and with +15.75 D spherical and +1.00 D at 42 degree cylindrical degree refractive errors in OS. Previously, he had clear lens cataract extraction to correct the refractive error in OD in another center. Axial lengths were 14.40 mm OD and 14.60 mm OS. Horizontal and vertical corneal diameters were 9.7 mm and 10.2 mm in right eye and 9.8 mm and 10.2 mm in left eye, respectively. The intraocular pressure was measured as 14 mmHg OD, 15 mmHg OS. Central corneal thicknesses of right and left eye were 538 microns and 544 microns, respectively. Temporal anterior chamber degrees of right and left eye were 36.19 degrees and 31.44 degrees, respectively. Fundus examination showed crowded discs with blurred margins along with horizontal macular folds emanating from the fovea and extending nasally to the optic disc (Figure 3). Horizontal and vertical papillary diameters of OD and OS were 2312 *μ*, 2209 *μ* and 1138 *μ*, 1442 *μ*, respectively. Choroidal thicknesses of OD and SO were 406 *μ* and 399 *μ*, respectively. OCT demonstrated papillomacular fold of sensorial retina without involvement of underlying retinal pigment epithelium and choroid (Figure 4).

## 4. Discussion

Various macular changes including papillomacular folds and macular radial folds have been described with nanophthalmic eyes [4]. Foveal avascular zone abnormalities in nanophthalmic eyes have been reported. Pigmentary retinal degeneration also rarely occurs with nanophthalmos [5]. Multiple fine macular retinal folds have been described in association with hyperopia by Cross and Yoder [6]. Large horizontal retinal folds involving the macula have been described as isolated congenital malformations associated with cataracts or with retrolental fibroplasias [7]. Uemura and Morizane reported six cases with bilateral high hyperopia and papillomacular retinal folds that had visual acuity ranged from 0.1 to 0.4 [8].

Our patients had axial lengths less than 16 mm; therefore crowded discs, yellowish reflex, and macular folds were seen because of the crowding of the posterior segment. Visual acuities of the patients were 0.3 and 0.3. Early detection of this condition is very important for the amblyopia therapy.

Vision may be affected in these patients due to high refractive amblyopia and/or structural macular changes. Papillomacular folds, which are common among patients with posterior microphthalmos, are also seen in patients with nanophthalmos. The OCT scans of patients with posterior microphthalmos and retinal folds have demonstrated that the neurosensory retina was folded; however the retinal pigment epithelium layer and choroid were intact without folding [9]. It is presumed that the retinal folds result from a redundancy of the retinal layer caused by retarded growth of the scleral and retinal pigment epithelium layers. It is possible that the same pathophysiology is observed in nanophthalmos and posterior microphthalmos since they are both in the spectrum of microphthalmos. The anatomic content of the macular folds could only be assessed by the OCT images. Only few reports showed the OCT analysis of these folds. All of the studies except the reports of Khan and Zafar and Timoney et al. demonstrated that neurosensory retina was folded with intact retinal pigment epithelium layer [2, 10]. Timoney et al. reported chorioretinal folds involving both the retina and choroid by OCT in two cases of nanophthalmos associated with Kenny-Caffey syndrome [10]. In the case series of Khan and Zafar elevated chorioretinal fold of normal thickness with an underlying empty vaulted area was reported to be seen in OCT. Our cases and other reports clearly demonstrated that papillomacular folds seen in nanophthalmos or posterior microphthalmos were only composed of neurosensory retina without involvement of retinal pigment epithelium and choroid; therefore they could be treated as serous retinal detachments. Although Kim et al. proposed that liquids heavier than water such as perfluorocarbon could be used to iron out these folds, there is not any surgical operation accepted for this condition yet [9].

## 5. Conclusion

Crowded posterior segment in nanophthalmic eyes may lead to retinal changes described in our cases. OCT clearly demonstrated that only neurosensory retina was involved in papillomacular folds in nanophthalmos. High refractive amblyopia and chorioretinal changes are the major causes of low vision in these patients.
